# Evaluation of Lipid Profile and Intima Media Thickness in Antiretroviral-Experienced HIV-Infected Patients Treated with Protease Inhibitor-Based Regimens versus Protease Inhibitor-Sparing Regimens

**DOI:** 10.3390/pathogens12070925

**Published:** 2023-07-10

**Authors:** Salvatore Martini, Mariantonietta Pisaturo, Antonio Russo, Maria Grazia Palamone, Maria Teresa Russo, Verdiana Zollo, Paolo Maggi, Nicola Coppola

**Affiliations:** Infectious Disease Unit, University of Campania Luigi Vanvitelli, L. Armanni 5, 80131 Naples, Italy; salvatoremartini76@gmail.com (S.M.); mariantonietta.pisaturo@unicampania.it (M.P.); antoniorusso.ar.ar@gmail.com (A.R.); mariagrazia.palamone@gmail.com (M.G.P.); terry93.mtr@gmail.com (M.T.R.); verdianazollo@gmail.com (V.Z.); paolo.maggi@unicampania.it (P.M.)

**Keywords:** HIV, PIs, IMT, dyslipidemia, CVD

## Abstract

Background: Antiretroviral therapy has increasingly improved management of HIV infection, ensuring long-term efficacy and tolerability. Each class of antiretrovirals has, however, different characteristics and different tolerability profiles. The literature data show that protease inhibitors (PIs) are associated with a higher incidence of dyslipidemia. The aim of our study was to evaluate whether patients treated with PIs have both greater dyslipidemia and increased intima media thickness (IMT) and atheromatous plaques compared to patients treated without PIs. Materials and Methods: A total of 110 HIV-experienced patients screened with Doppler ultrasonography of the supra-aortic trunks in December 2019 were enrolled in a retrospective cross-sectional observational study. Patients were divided into two groups: 59 in the PI-based group, treated with PIs, and 51 in the PI-sparing group. In the two groups, we evaluated lipids, cardiovascular risk factors (smoking, BMI, age, hypertension), increased pathological IMT (a value > 1 mm), and possible atheromatous plaque. Results: Serum LDL (*p* 0.04) and percentage of patients with hypercholesterolemia (*p* 0.03) were higher in the PI-based than in the PI-sparing group. Doppler data showed a trend in increase of IMT > 1 in the PI-based group, which appeared statistically significant for the section of the left common carotid artery (*p* 0.03). However, in multivariate logistic regression models, none of the evaluated variables were significantly associated with IMT > 1. Conclusions: Our real-life data show that patients treated with PIs have a trend of developing both greater dyslipidemia and increased pathological IMT and atheromatous plaques These findings may be useful to optimize antiretrovirals for patients with cardiovascular risk factors.

## 1. Introduction

The advent of protease inhibitors (PIs) has strongly changed the prognosis of patients with human immunodeficiency virus (HIV) infection. In fact, their high genetic barrier obtains long-term efficacy in the control of viral replication. However, treatment with PIs may induce dyslipidemia, which may be a problem for patients who become aged, who may have weight gain, and who may smoke and may develop atheromatous plaques [1], and thus with a higher risk of cardiovascular disease (CVD) [2,3]. Therefore, it is possible to hypothesize that PIs may be related to an increase of intima media thickness (IMT), evaluated with Doppler ultrasonography of the supra-aortic trunks [4,5,6]. 

CVD is one of the most important causes of mortality and morbidity in the industrialized world. People with hyper-lipidemia, in fact, are at roughly twice the risk of developing CVD compared to those with normal total cholesterol levels [7]. In particular, hypercholesterolemia and especially elevated levels of low-density lipoprotein cholesterol (LDL-C) are the most important risk factors that contribute to the formation and progression of atherosclerotic plaques [8]. People living with HIV (PLWHIV) have a two-fold higher risk of having a cardiovascular event than HIV-negative individuals [9,10]. However, in the literature there are discordant data on the role of PI-based regimens in the onset of IMT. 

In the present study, we evaluated PLWHIV, treated in real life with different types of regimens and different PIs, used in triple or dual therapy, compared to PI-sparing regimens, including non-nucleoside reverse-transcriptase inhibitors (NNRTIs) and integrase inhibitors (INIs), and screened in December 2019 by Doppler ultrasonography of the supra-aortic trunks. The aim of our study was to analyze the possible association between the use of PIs in antiretroviral treatment, in any regimen, and the associated damage at the endothelium of supra-aortic trunks, expressed as pathological IMT and/or atheromatous plaques. 

## 2. Patients and Methods

### 2.1. Study Design

We performed an observational, retrospective, cross-sectional study enrolling 110 HIV-experienced patients who were consecutively screened with Doppler ultrasonography of the supra-aortic trunks in December 2019 and treated with the same antiretroviral regimen. Patients were divided into 2 groups: 59 were treated with a PI-based antiretroviral regimen (PI-based group) and 51 were treated with an antiretroviral regimen without PIs (PI-sparing group). 

In the 2 groups of subjects, we evaluated the following parameters:

1. IMT of common and internal carotid for both left and right sides: ultrasonography of the epi-aortic vessels using a power color-Doppler instrument with 7.5 MHz probes. We evaluated the characteristics of the intima, together with the pulsation index, resistance index, minimal speed, peak speed and mean speed. A minimum of three measurements were required: on the common carotid artery, 1 cm before the carotid bifurcation and at the carotid bifurcation; on the internal carotid, 1 cm after the carotid bifurcation and 2 cm after the carotid bifurcation. The definition of IMT is the distance between the leading edge of the media–adventitia echo and the leading edge of the internal lumen. The larger IMT of the bilateral common carotid artery is the final value of the IMT. An IMT greater than 1 mm and/or atherosclerotic plaques are considered pathologic findings [11]. Atherosclerotic plaques, if present, were described. All images were photographed and archived.
2. Data regarding independent risk factors for cardiovascular disease (CVD), such as family history, smoking, and diabetes.
3. HIV viral load, CD4+ cell counts, total serum cholesterol, low-density lipoprotein (LDL) cholesterol, high-density lipoprotein (HDL) cholesterol, glycemia, triglycerides, and body mass index (BMI) were recorded at each control visit.

All procedures performed in this study were in accordance with the 1964 Helsinki declaration and its later amendments or comparable ethics standards. Informed consent was obtained from all participants included in the study.

### 2.2. Study Outcomes

The primary outcome was the evaluation of the development of pathologic IMT and its correlation with the PI-based regimen in HIV-experienced patients. The secondary outcome was the evaluation of the association between dyslipidemia and PI-based treatment. Dyslipidemia referred to unhealthy levels of one or more kinds of lipids in the blood (triglycerides > 150 mg/dL, cholesterol > 200 mg/dL, LDL > 115 mg/dL) and/or use of statins. We also evaluated cardiovascular risk index (CRI), which was calculated by dividing total cholesterol level by HDL level: an increased CRI was considered for values > 5 in men and >4.5 in women [12]. 

### 2.3. Serological Analysis

The HIV viral load was assessed by real-time PCR with the lowest detection limit of 40 copies/mL.

Lymphocyte subsets (CD4+, CD8+) were evaluated with flow cytofluorimetry using monoclonal antibodies and a fluorescence-activated cell sorter scan (Becton Dickinson, Mountain View, CA, USA). Serum lipids and routine analyses were performed applying standard procedures. 

### 2.4. Statistical Analysis

Continuous variables were summarized as mean and standard deviation or median at 25° and 75° quartile, and categorical variables as absolute and relative frequencies. To evaluate distribution of continuous variable we used the Shapiro–Wilk test, and to assess homoscedasticity we used Levene’s Test. For continuous variables, if normally distributed, the differences were evaluated by the Student’s *t*-test for comparison between two groups; in the case of non-normally distributed variables, the Mann–Whitney U test was performed; categorical variables were compared using the Chi-square or Fisher’s exact test. 

We performed univariate and multivariate logistic regression models to investigate possible association between IMT > 1 and different assessed parameters. Multivariate regression models were adjusted for age, BMI, diabetes, ARV history, and PI-based regimen. Results of the logistic regressions are presented as odds ratio (OR) and 95% confidence interval (CI). A *p*-value below 0.05 was considered statistically significant. Analyses were performed using STATA (version STATAC 16.1).

## 3. Results

The characteristics at enrollment of the 110 PLWHIV included in the study are summarized in Table 1. The median age of the patients was 52 years (Q1–Q3 47–60), 77.3% were males, 88% were Italian subjects, 39% were men who have sex with men (MSM) and 42.7% had unsafe heterosexual intercourse as a risk factor for HIV infection. Seven (6.4%) subjects were in the CDC-C stage, while the CD4 nadir cell count mean was 293.5 (Q1–Q3 166–439). At enrolment, 38.5% of subjects showed hypertriglyceridemia, 29.4% hypercholesterolemia, and 25.7% high serum value of LDL (Table 1). 

Of the 110 HIV-experienced patients enrolled in the study, 59 were included in the PI-based group, and 51 in the PI-sparing group. The subjects in the PI-based group were treated with PI for a median of 96 months, a mean of 103 (±26.2 standard deviation, SD), while those in the PI-sparing group were treated on antiretroviral therapy for a median of 96 months, a mean of 111.6 (±61.44 SD). Specifically, as regards the type of drugs used, in the PI-sparing group 13 patients were treated with integrase inhibitors (INIs) [2 (15.4%) with raltegravir (RAL), 8 (61.5%) with dolutegravir (DGT), and 3 (23%) with elvitegravir/cobicistat (EVG/c)]; 38 were treated with NNRTIs [8 (21%) with nevirapine (NVP), 12 (31.6%) with efavirenz (EFV), and 18 (47.36%) with rilpivirine (RPV)]. In the PI-based group, 46 patients were treated with a triple-therapy regimen containing PI [8 (17.3%) with lopinavir/ritonavir (LPV/r), 25 (54.3%) with darunavir/ritonavir (DRV/r), 8 (17.3%) with atazanavir/ritonavir (ATV/r), 4 (8.7%) with unboosted ATV, and 1 (2.1%) with saquinavir/ritonavir (SQV/r)]. In the same group, 13 patients were treated with PI in dual therapy [2 (15.3%) with RAL + LPV/r, 6 (46.1%) with RAL + DRV/r, 3 (23%) with DGT + DRV/r, 1 (7.6%) with RAL + ATV], while 1 (7.6%) was treated with a different dual regimen associating NNRTI (Etravirine) and DRV/r. The backbone of ART was similar between the 51 subjects in the PI-based group and the 46 in the PI-based group in triple ART therapy.

There were no significant differences in the demographic, clinical, biochemical, and viro-immunological data between the two groups, although the median age was higher in the PI group (*p* 0.002) (Table 1). In the PI-based group, moreover, patients had a longer history of antiretroviral therapy (*p* 0.003) (Table 1), but univariate and multivariate analysis showed no significant association of this data with higher IMT (Table 2).

All lipid parameters (triglycerides, cholesterol, LDL) were similar in the two groups, except for median serum LDL (*p* 0.04), and the percentage of patients with hypercholesterolemia (*p* 0.03) was higher in the PI group (Table 1). As regards CRI, no pathological increase was observed in either group.

**Figure 1:**
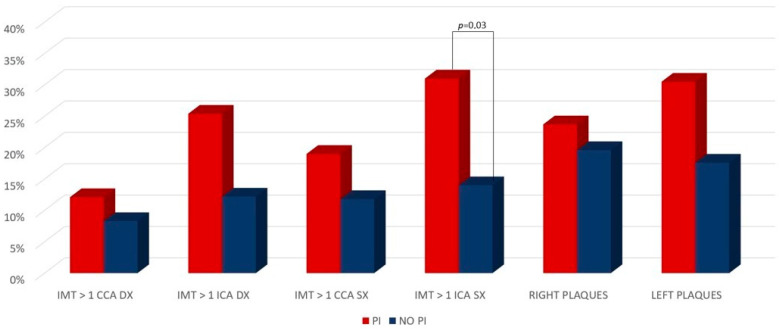
IMT and atheromatous plaques in the PI-based and PI-sparing groups.

In terms of the primary outcome, Doppler data showed a trend in the increase of IMT > 1 in the PI-based group, which appeared statistically significant for the section of the left common carotid artery (CCA) (*p* 0.03) (Table 3 and Figure 1). The demographic, clinical, biochemical, and viro-immunological data associated with IMT > 1 mm are shown in Table 3. After performing multivariate logistic regression models, none of the evaluated variables were significantly associated with IMT > 1 (Table 2).

## 4. Discussion

The advent of PIs has given HIV patients the hope of surviving but at the risk of developing some comorbidities related to drugs and/or to aging. Therefore, this prognostic improvement has forced clinicians to manage different comorbidities and above all long-term metabolic status. PIs in particular appear related to higher risk of dyslipidemia, which is a cardiovascular risk factor above all when it is associated with the development of atherosclerotic plaques in the supra-aortic trunks [1]. 

We sought to demonstrate that there may be an association between PIs and cardiovascular risk with the evidence of dyslipidemia associated with a higher rate of pathological IMT. This alteration is preparatory to the development of atherosclerosis and CVD. Comparing Doppler data of patients treated with PIs vs. those without PIs, we highlighted that there is a correlation between these drugs and elevated IMT. This was statistically significant in particular for the section of the left internal carotid artery (ICA). 

The data in the literature on the association between PIs and CVD are conflicting. In 2005, Iloeie et al., evaluating 7542 patients, observed that patients exposed to PI therapy had an increased risk of CVD events, and therefore clinicians should evaluate the risk of CVD when making treatment decisions for HIV-infected patients [13]. In reverse, in a paper of 2007, Lebech et al., enrolling non-smoking HIV patients, found no correlation between PIs and IMT [14]. In addition, hypercholesterolemia was not related to IMT, which was evident only inversely with HDL cholesterol levels. Another study published in the same period, analyzing a cohort of 133 patients from eight medical centers in the United States, concluded that HIV infection and PI use did not contribute substantially to the rate of carotid IMT progression [15]. On the contrary, two years later, Sankatsing et al., in their work showed an increase of IMT in patients treated for 2 years with PIs versus patients treated with NNRTIs [16]. These data appear controversial, but each case was related to old regimens, based on old PIs. Currently, the only PI used in clinical practice is darunavir. In the present paper, more than 50% of the subjects in the PI-based group had been in ART for at least 6 months with darunavir/ritonavir. A more recent paper, evaluating data from Janssen-sponsored clinical trials, suggests that CVD should not be considered an important risk for users of darunavir/ritonavir [17]. In another study, Brazilian authors enrolled 40 patients using PIs, 40 not using Pis, and 65 controls to evaluate differences in IMT. They concluded that IMT of the carotid arteries was greater in PLWHIV and occurred earlier compared with controls, regardless of the use of PIs [18]. 

As regards the pathogenetic mechanisms of this association, some authors have hypothesized that the ART activates endothelial function and promotes atherosclerosis. It is possible that HIV, immune reconstitution response, and ART may promote early endothelial activation, and hence represent pro-atherogenic factors and/or accelerators of atherosclerosis [19,20]. Analyzing data from the literature, it appears that recent papers, in contrast to the older ones, seem to assign more importance to the role of HIV infection and PI-based regimen in the onset of higher IMT. In a recent work in 2021, Ounjaijean et al. showed, in an Asiatic cohort, that there was higher cardiovascular risk in HIV patients receiving PI-based regimens [21].

The increased risk of atherosclerotic cardiovascular disease among HIV-infected patients receiving a PI-based regimen may not be directly due to the acceleration of the pro-inflammatory response. The major mechanism in which PI-based regimens were triggering atherogenesis could be through an alteration of lipid metabolism and endothelial function. In a recent review in 2021, Vos et al. concluded that PIs and efavirenz have associated metabolic disturbances and increased risk of CVD, although their use was decreasing worldwide [22]. In 2014, Reyskens and Essop [23] analyzed the role of PI in the onset of cardio-metabolic complications and investigated the relationship between oxidative stress and the ubiquitin–proteasome system (UPS) as key mediators driving this process.

The authors hypothesized that PIs trigger reactive oxygen species (ROS) production, which leads to serious downstream consequences such as cell death, impaired mitochondrial function, and UPS dysregulation. HIV PI-induced ROS, together with a dysfunctional UPS, elicit definitive effects on the cardiovascular system that will eventually result in the onset of heart diseases. Thus, while PIs substantially improve life expectancy and quality of life in HIV-positive patients, their longer-term side-effects on the cardiovascular system should lead to (a) greater clinical awareness regarding the benefit–harm paradigm, and (b) the development and evaluation of novel co-treatment strategies. Analyzing data from the literature, it appears that recent papers, in contrast to the older ones, seem to give more importance to the role of HIV infection and PI-based regimens in the onset of higher IMT. These data may suggest that CVD is a risk factor related to the class of PI and to specific previous drugs. In fact, in a recent review of 2021, Hateleberg et al. concluded that although PIs will likely remain in use globally for several years to come, baseline CVD risk should be considered when selecting their use, especially as the population with HIV ages [24]. 

This is the reason why more metabolic-friendly treatment regimens have been developed in recent years. The advent of INIs has allowed the adoption of two-drug regimens, with similar efficacy and a better tolerability profile than triple regimens [25,26,27,28,29].

This study has some limitations, being a retrospective cross-sectional monocenter design, and moreover, the study was concluded in December 2019 and consequently no patient had been treated with bictegravir because this drug was not available at that time. There were 14 patients in the PI-sparing group with a previous history of PI-based therapy, but they represented only 27% of the group and the average duration of therapy with PI was only 18 months, probably insufficient to cause tangible endothelial damage.

Furthermore, in the PI group, the authors used four different PI/r and an unboosted PI in five cases. There may be a potentially different cardiovascular risk associated with the different PIs adopted. For example, ATV was known to be a PI with lower metabolic impact and lower cardiovascular risk than other PIs [30], especially if unboosted. In the PI group, however, only 13 (22%) of 59 patients were treated with ATV (8 ATV/r and 5 unboosted ATV); moreover, we was not able to identify the impact of the different PIs on the development of cardiovascular diseases. However, the intent of the study is to evaluate the cardiovascular risk related altogether to the class of PIs, which was also highlighted in a recent review of Batta et al. [31].

In conclusion, observing PLWHIV treated in real life, we noted more pathological IMT in patients treated with PI vs. PI-sparing regimens. This may be related to a higher risk of CVD in these patients, as shown in the literature. It is possible that endothelial damage is progressive, and in our study, patients in the PI group had been treated for longer than the PI-sparing group. In any case, this may further support the possibility of a proactive switch from PI to other drugs with a more metabolic-friendly profile for patients with a potential high risk of CVD, such as diabetics and the elderly.

## Figures and Tables

**Table 1:** Demographic, clinical, biochemical, and viro-immunological data at enrollment.

Data at Baseline	Total	PI-Sparing	PI-Based	*p*
Number of patients	110	51	59	
Median age (Q1–Q3)	52 (47–60)	50 (45–55)	56 (50–61)	0.002
Male (%)	85 (77.3)	41 (80.4)	44 (74.6)	0.468
Italians (%)	97 (88.1%)	43 (84.3%)	54 (91.5%)	0.38
Risk Factors for HIV infection	MSM 43 (39%)	MSM 23 (45%)	MSM 20 (33.89%)	0.31
	PWID 20 (18.18%)	PWID 5 (9.80%)	PWID 15 (25.42%)	0.06
	HETERO 47 (42.72%)	HETERO 23 (45%)	HETERO 24 (40.67%)	0.78
Patients with BMI > 29 (%)	12 (10.9)	7 (13.7)	5 (8.5)	0.378
Patients with a history of PI-based treatment (%)	73 (66.36)	14 (27.4)	59 (100)	0.001
Median total years of ART (Q1–Q3)	9 (7–15)	8 (5–10)	10 (8–16.26)	0.003
Median months of previous PI-based treatment (Q1–Q3)	84 (0–120)	0 (0–10)	96 (84–120)	0.000
Patients treated with statins (%)	16 (15%)	4 (7.8)	12 (21.4)	0.049
Patients with other lipid-lowering drugs (%)	23 (20.9)	6 (11.7)	17 (28.8)	0.09
Hypertensive patients (%)	30 (27.5)	13 (25.5)	17 (29.3)	0.656
Patients treated with anti-hypertensives (%)	29 (28.2)	13 (28.3)	16 (28.1)	0.983
Diabetic patients (%)	8 (7.4)	5 (9.8)	3 (5.3)	0.368
Smokers (%)	45 (47.4)	17 (37.8)	28 (56)	0.076
CDC Classification: Class C (late presenters) (%)	7 (6.4)	3 (5.9)	4 (6.8)	0.823
Median CD4+ cell/µL (Q1–Q3)	658 (484–871)	643 (460–890)	676 (489–851)	0.861
Median CD4+ NADIR, cell/µL (Q1–Q3)	293.5 (166–439)	308 (199–447)	244 (101–429)	0.158
Median triglycerides (mg/dL) (Q1–Q3)	118 (85–179)	98 (81–179)	98 (81–179)	0.052
Median cholesterol (mg/dL) (Q1–Q3)	180 (161–205)	172 (155–199)	188 (169–206)	0.095
Median HDL (mg/dL) (Q1–Q3)	45 (38–54)	47 (40–59	44 (37–51)	0.452
Median LDL (mg/dL) (Q1–Q3)	115 (97–134)	107 (91–126)	121 (106–141)	0.043
Patients with triglycerides > 150 mg/dL (%)	42 (38.5)	16 (31.4)	26 (44.8)	0.150
Patients with cholesterol > 200 mg/dL (%)	32 (29.4)	10 (19.6)	22 (37.9)	0.036
Patients with HDL < 40 mg/dL (%)	36 (33)	15 (29.4)	21 (36.2)	0.088
Patients with LDL > 115 mg/dL (%)	53 (48.1)	20 (39.2)	33 (55.9)	0.11

Footnotes: HDL (High-Density Lipoprotein), LDL (Low-Density Lipoprotein), BMI (Body Mass Index), PI (Protease Inhibitor), ART (AntiRetroviral Therapy), MSM (Men who have Sex with Men), PWID (People Who Inject Drugs), HETERO (Heterosexual).

**Table 2:** Univariate and multivariate analysis of demographic, clinical, biochemical, and viro-immunological data associated with IMT > 1 mm.

Data at Baseline	IMT ICA SX < 1.01	IMT ICA SX ≥ 1.01	Univariate Analysis	Multivariable Analysis		
			*p* Value	OR	95% Confidence Interval	*p* Value
Number of patients	83	27	NA			
Median age (Q1–Q3)	52 (46–60)	53 (50–58)	0.543	1.006	0.958–1.055	0.822
N° of males (%)	64 (77.1)	19 (76)	0.908	NA	NA	NA
N° of patients with BMI > 29 (%)	11 (13.3)	1 (4)	0.197	1.028	0.247–4.273	0.969
N° of patients treated with PIs	40 (48.3)	18 (72)	0.04	1.115	0.418–2.978	0.828
N° of patients with previous PI-based regimen (%)	32 (39)	6 (24)	0.169	NA	NA	NA
Median total years of antiretroviral treatment (Q1–Q3)	9 (7–13)	9.01 (8–15.78)	0.224	0.933	0.844–1.033	0.171
Median months of previous treatment with PIs (Q1–Q3)	84 (0–96)	84 (60–120)	0.125	NA	NA	NA
N° of patients treated with statin (%)	8 (9.9)	8 (33.3)	0.005	NA	NA	NA
N° of patients treated with other lipid-lowering drugs	14 (16.86)	9 (36)	0.04	NA	NA	NA
N° of patients treated with antihypertensive drugs (%)	17 (22.4)	10 (40)	0.084	NA	NA	NA
N° of diabetic patients (%)	3 (3.7)	4 (16)	0.030	1.322	0.234–7.475	0.752
N° of smoker patients (%)	32 (44.4)	12 (48)	0.305	NA	NA	NA
CDC CLASSIFICATION: Class C (late presenters) (%)	4 (4.8)	3 (12)	0.497	NA	NA	NA
Median CD4+ cell count (Q1–Q3)	688 (502–890)	633 (489–736)	0.466	NA	NA	NA
Median CD4+ NADIR, cell/µL (Q1–Q3)	300.5 (184–447)	241 (94–439)	0.405	NA	NA	NA
Median triglycerides (mg/dL) (Q1–Q3)	120 (84–194)	117 (98–169)	0.724	NA	NA	NA
Median cholesterol (mg/dL) (Q1–Q3)	182 (155–207)	180 (171–199)	0.752	NA	NA	NA
Median HDL (mg/dL) (Q1–Q3)	46 (38–55)	44 (40–48)	0.699	NA	NA	NA
Median LDL (mg/dL) (Q1–Q3)	116 (95–139)	115 (106–126)	0.724	NA	NA	NA
N° of patients with triglycerides >150 mg/dL (%)	32 (39%)	10 (40)	0.930	NA	NA	NA
N° of patients with cholesterol > 200 mg/dL (%)	26 (31.7)	6 (24)	0.461	NA	NA	NA
N° of patients with HDL < 40 mg/dL (%)	28 (34.1)	7 (28)	0.566	NA	NA	NA
N° of patients with LDL > 115 mg/dL (%)	42 (50.6)	12 (44.4)	0.73	NA	NA	NA

Footnotes: IMT (Intima–Media Thickness), HDL (High-Density Lipoprotein), LDL (Low-Density Lipoprotein), BMI (Body Mass Index), PIs (Protease Inhibitors), ICA (Internal Carotid Artery).

**Table 3:** Doppler TSA data according to groups of subjects.

	Total	PI-Sparing	PI-Based	*p*
Number of patients	110	51	59	
IMT > 1 CCA DX (%)	11 (10.4)	4 (8.3)	7 (12.1)	0.530
IMT > 1 ICA DX (%)	21 (19.1)	6 (12.2)	15 (25.4)	0.085
IMT > 1 CCA SX (%)	17 (15.4)	6 (11.8)	11 (19)	0.301
IMT > 1 ICA SX (%)	25 (22.7)	7 (14)	18 (31)	0.036
TOTAL PLAQUES	51 (46.3%)	19 (37.25%)	32 (54.23%)	0.11
TOTAL RIGHT PLAQUES (%)	24 (21.8)	10 (19.6)	14 (23.7)	0.602
TOTAL LEFT PLAQUES (%)	27 (24.5)	9 (17.6)	18 (30.5)	0.118

Footnotes: IMT (Intima–Media Thickness), CCA (Common Carotid Artery), ICA (Internal Carotid Artery), PI (Protease Inhibitor).

## Data Availability

The data can be requested from the corresponding author, Nicola Coppola (nicola.coppola@unicampania.it).
